# 4,6-Bis(diphenyl­phosphan­yl)-2,8-di­methyl­phenoxathiin dichloro­methane monosolvate

**DOI:** 10.1107/S1600536812009920

**Published:** 2012-03-10

**Authors:** Thashree Marimuthu, Holger B. Friedrich, Muhammad D. Bala

**Affiliations:** a School of Chemistry, University of KwaZulu-Natal, Westville Campus, Private Bag X54001, Durban 4000, South Africa

## Abstract

The title compound, C_38_H_30_OP_2_S·CH_2_Cl_2_, belongs to the xanthene family of ligands containing S- and O-donor atoms in the central heterocylic ring. Positions 2 and 8 on the xanthene backbone are functionalized with methyl groups to allow for the selective functionalization of the backbone at positions 4 and 6 with diphenyl­phosphanyl units. The title compound shows a significant ‘roof-like’ bending along the axis of planarity involving the O- and S-donor atoms and the benzene rings, resulting in a dihedral angle between the mean planes of the benzene rings of 32.88 (13)°.

## Related literature

For a closely related compound, see: Goertz *et al.* (1998 ▶). For complexation to metal centre and catalysis, see: Kranenburg *et al.* (1995 ▶). For related P-donor ligands, see: Marimuthu *et al.* (2008 ▶). For a related structure, see: Hillebrand *et al.* (1995 ▶).
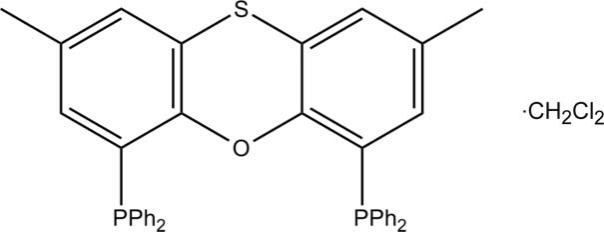


## Experimental

### 

#### Crystal data

C_38_H_30_OP_2_S·CH_2_Cl_2_
*M* *_r_* = 681.55
Monoclinic, 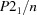

*a* = 9.3605 (7) Å
*b* = 20.6796 (15) Å
*c* = 18.1360 (15) Å
β = 104.955 (1)°
*V* = 3391.7 (5) Å^3^
*Z* = 4
Mo *K*α radiation
μ = 0.38 mm^−1^
*T* = 173 K
0.47 × 0.36 × 0.28 mm

#### Data collection

Bruker APEXII CCD diffractometer
46059 measured reflections
8183 independent reflections
6527 reflections with *I* > 2σ(*I*)
*R*_int_ = 0.039

#### Refinement

*R*[*F* ^2^ > 2σ(*F* ^2^)] = 0.036
*wR*(*F* ^2^) = 0.100
*S* = 1.07
8183 reflections
408 parameters
H-atom parameters constrained
Δρ_max_ = 0.46 e Å^−3^
Δρ_min_ = −0.51 e Å^−3^

### 

Data collection: *APEX2* (Bruker, 2005 ▶); cell refinement: *SAINT-Plus* (Bruker, 2005 ▶); data reduction: *SAINT-Plus*; program(s) used to solve structure: *SHELXS97* (Sheldrick, 2008 ▶); program(s) used to refine structure: *SHELXL97* (Sheldrick, 2008 ▶); molecular graphics: *SHELXTL* (Sheldrick, 2008 ▶); software used to prepare material for publication: *SHELXTL*.

## Supplementary Material

Crystal structure: contains datablock(s) I, global. DOI: 10.1107/S1600536812009920/fj2525sup1.cif

Structure factors: contains datablock(s) I. DOI: 10.1107/S1600536812009920/fj2525Isup2.hkl

Supplementary material file. DOI: 10.1107/S1600536812009920/fj2525Isup3.cml

Additional supplementary materials:  crystallographic information; 3D view; checkCIF report

## Figures and Tables

**Fig. 1.**
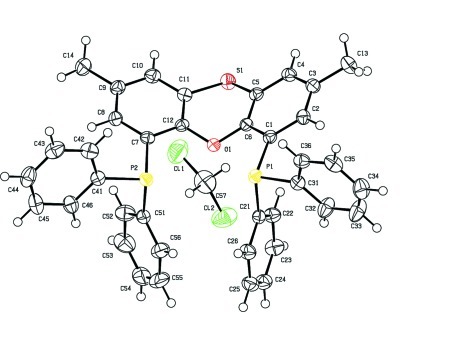
ORTEP diagram of compound (I). Thermal ellipsoids are represented at the 50% probability level

## supplementary crystallographic information

### Comment

The title compound, 4,6-bis(diphenylphosphanyl)-2,8-dimethylphenoxathiin (I) (Fig. 1), has been reported as a ligand on rhodium for hydroformylation of olefins and on nickel for hydrocyanation of styrene (Kranenburg *et al.*, 1995). Compound (I) consists of two very nearly regular planar hexagonal carbocyclic rings joined to a non-planar heterocyclic ring. The planes of the aromatic rings of the xanthene backbone bisect at an angle of 147.12° with the S atom lying out of the ring planes.

A comparison of (I) with the closely related structure reported by Goertz *et al*. (1998) shows that sixantphos can adopt two different conformations in the solid state with different crystallographic parameters and cell contents. The conformational differences were due to the presence of an incorporated solvent molecule in (I) and the significant roof-like bending of the backbone framework along the axis of planarity involving the O and S heterocyclic atoms and the aromatic rings. The dihedral angle of 32.88° between the least-squares planes of the aromatic rings of (I) is significant when compared to the essentially co-planar aromatic rings in the phenoxazine backbone of the Goertz *et al.* (1998) compound. The title compound crystallizes in the *P*2_1_/*n* space group, while the previously reported structure was in the *P*2_1_/*c* space group. A similar difference in conformation was reported by (Hillebrand *et al.*, 1995) for two separate crystals of xantphos. The bond lengths for (I) are in good agreement with those in the reported structure, but the internal bond angles of the heterocyclic ring are slightly shorter in (I). This is consistent with a bent geometry of the heterocyle in (I) [C11—S1—C5] 98.28 (7) and [C12—O1—C6] 117.5 (1)° compared to 101.4 (1) and 124.4 (2)° for similar bond angles in the previously reported crystal structure.

Upon complexation to a metal centre the backbone of sixantphos tends to bend to accommodate the extra steric congestion around the metal centre (Goertz *et al.*, 1998). Therefore when compound (I) is used as a ligand, the backbone needs little tilting in order to coordinate to a metal centre. The bond angles at the P atoms range from 100.04 (6) to 102.95 (7)° which are similar to those found for a realated P donor ligand [99.93 (10) to 103.02 (10)°] (Marimuthu *et al.*, 2008).

### Experimental

A solution of 2,8-dimethylphenoxathiin (1.5 g, 6.6 mmol) and TMEDA (2.5 ml, 16.8 mmol) in 45 ml of dry degassed Et_2_O was cooled to 0 °C. To the chilled solution, *n*BuLi (10.3 ml, 16.8 mmol) was added dropwise. The reaction mixture was allowed to warm to room temperature and left to stir for 16 h. The resulting dark orange reaction mixture was cooled to 0 °C and PPh_2_Cl (3.1 ml, 16.8 mmol) in hexane (6 ml) added dropwise. The reaction mixture slowly decolourized and a fine precipitate formed. The reaction was allowed to stir for a further 16 h. Thereafter, the reaction was slowly hydroylsed with 40 ml of 10% HCl/brine mixture (1/1). The organic layer was removed, and the aqueous layer extracted with dichloromethane. The combined fractions were dried over MgSO_4_, filtered, and the volume reduced to give a yellow oil. The crude product was washed with hexane (3 *x* 20 ml), the oil dissolved in dichloromethane, and an equal volume of ethanol added slowly. The solution was left to recrystalize at room temperature and the crystals filtered and dried under vacuum. Recrystallization from dichloromethane/ethanol (1:1) afforded colourless crystals of (I) suitable for X-ray analysis. [yield: 2.2 g, 62%; m.p. 457 K]. Spectroscopic analysis: ^1^H NMR (400 MHz, CDCl_3_, δ, p.p.m.): 7.29 – 7.12 (m, 20H), 6.86 (apparent d, *J* = 1.0 Hz, 2H), 6.22 (bs, 2H), 2.05 (s, 6H); ^13^C NMR (101 MHz, CDCl_3_, δ, p.p.m.): 152.2(t, *J*(P,*C*) = 24.4 Hz, CO), 137.2 (t, *J*(P,*C*) = 13.1 Hz phenyl C-*ipso*, PC), 133.9 (t, *J* (P,*C*) =21 Hz, CH phenyl), 133.5 (C), 132.7 (CH), 128.2 (CH phenyl), 128.1 (t, *J*(P,*C*) = 3.5 Hz, CH phenyl), 127.6 (CH) 127.3 (dd, *J*(P,*C*) = 12.6, 11 Hz, CHCH*C*–P), 119.5 (CS), 20.6 (CH_3_); ^31^P NMR (243 MHz, CDCl_3_δ, p.p.m.): -17.9; IR (neat, ν_max_, cm-1): 3050 (w), 2961 (w), 2921 (w), 1556 (*m*), 1476 (*m*), 1432 (*m*), 1402 (*s*), 1238 (*m*), 1221 (*m*) 1199 (*m*), 742 (*s*), 692 (*s*); HR—MS (ESI) (m/*z*): 597.1559 [*M* + H]^+^ calcd. for C_38_H_31_OP_2_S, 597.1565.

### Refinement

All H-atoms were refined using a riding model, with C—H = 0.95 Å and *U*_iso_(H) = 1.2*U*_eq_(C) for aromatic and C—H = 0.98 Å and *U*_iso_(H) = 1.5*U*_eq_(C) for CH_3_.

### Crystal data

**Table:**

C_38_H_30_OP_2_S·CH_2_Cl_2_	*F*(000) = 1416
*M**_r_* = 681.55	*D*_x_ = 1.335 Mg m^−^^3^
Monoclinic, *P*2_1_/*n*	Mo *K*α radiation, λ = 0.71073 Å
Hall symbol: -P 2yn	Cell parameters from 9922 reflections
*a* = 9.3605 (7) Å	θ = 2.3–28.3°
*b* = 20.6796 (15) Å	µ = 0.38 mm^−^^1^
*c* = 18.1360 (15) Å	*T* = 173 K
β = 104.955 (1)°	Prism, colourless
*V* = 3391.7 (5) Å^3^	0.47 × 0.36 × 0.28 mm
*Z* = 4	

### Data collection

**Table:**

Bruker APEXII CCD diffractometer	6527 reflections with *I* > 2σ(*I*)
Radiation source: fine-focus sealed tube	*R*_int_ = 0.039
Graphite monochromator	θ_max_ = 28.0°, θ_min_ = 1.5°
φ and ω scans	*h* = −12→12
46059 measured reflections	*k* = −27→27
8183 independent reflections	*l* = −15→23

### Refinement

**Table:**

Refinement on *F*^2^	Primary atom site location: structure-invariant direct methods
Least-squares matrix: full	Secondary atom site location: difference Fourier map
*R*[*F*^2^ > 2σ(*F*^2^)] = 0.036	Hydrogen site location: inferred from neighbouring sites
*wR*(*F*^2^) = 0.100	H-atom parameters constrained
*S* = 1.07	*w* = 1/[σ^2^(*F*_o_^2^) + (0.0535*P*)^2^ + 0.4429*P*] where *P* = (*F*_o_^2^ + 2*F*_c_^2^)/3
8183 reflections	(Δ/σ)_max_ = 0.001
408 parameters	Δρ_max_ = 0.46 e Å^−^^3^
0 restraints	Δρ_min_ = −0.51 e Å^−^^3^

### Special details

**Table:**

Geometry. All e.s.d.'s (except the e.s.d. in the dihedral angle between two l.s. planes) are estimated using the full covariance matrix. The cell e.s.d.'s are taken into account individually in the estimation of e.s.d.'s in distances, angles and torsion angles; correlations between e.s.d.'s in cell parameters are only used when they are defined by crystal symmetry. An approximate (isotropic) treatment of cell e.s.d.'s is used for estimating e.s.d.'s involving l.s. planes.
Refinement. Refinement of *F*^2^ against ALL reflections. The weighted *R*-factor *wR* and goodness of fit *S* are based on *F*^2^, conventional *R*-factors *R* are based on *F*, with *F* set to zero for negative *F*^2^. The threshold expression of *F*^2^ > σ(*F*^2^) is used only for calculating *R*-factors(gt) *etc*. and is not relevant to the choice of reflections for refinement. *R*-factors based on *F*^2^ are statistically about twice as large as those based on *F*, and *R*- factors based on ALL data will be even larger.

### Fractional atomic coordinates and isotropic or equivalent isotropic displacement parameters (Å^2^)

**Table:**

	*x*	*y*	*z*	*U*_iso_*/*U*_eq_	
C1	0.67489 (15)	−0.01794 (7)	0.12353 (8)	0.0246 (3)	
C2	0.61337 (16)	−0.07280 (7)	0.08142 (8)	0.0275 (3)	
H2	0.6780	−0.1031	0.0675	0.033*	
C3	0.46158 (16)	−0.08469 (7)	0.05916 (8)	0.0282 (3)	
C4	0.36781 (16)	−0.04140 (7)	0.08135 (8)	0.0274 (3)	
H4	0.2641	−0.0486	0.0671	0.033*	
C5	0.42542 (15)	0.01272 (7)	0.12445 (8)	0.0245 (3)	
C6	0.57712 (15)	0.02502 (7)	0.14406 (8)	0.0234 (3)	
C7	0.63835 (15)	0.19416 (7)	0.18215 (8)	0.0240 (3)	
C8	0.55781 (16)	0.25217 (7)	0.17171 (8)	0.0272 (3)	
H8	0.6101	0.2920	0.1770	0.033*	
C9	0.40319 (16)	0.25350 (7)	0.15383 (9)	0.0295 (3)	
C10	0.32755 (16)	0.19526 (7)	0.14747 (9)	0.0302 (3)	
H10	0.2225	0.1952	0.1351	0.036*	
C11	0.40387 (16)	0.13687 (7)	0.15907 (8)	0.0260 (3)	
C12	0.55822 (15)	0.13654 (7)	0.17502 (7)	0.0233 (3)	
C13	0.39974 (19)	−0.14374 (8)	0.01255 (10)	0.0377 (4)	
H13A	0.3703	−0.1759	0.0455	0.057*	
H13B	0.4754	−0.1623	−0.0097	0.057*	
H13C	0.3134	−0.1313	−0.0284	0.057*	
C14	0.31804 (19)	0.31613 (8)	0.14184 (11)	0.0410 (4)	
H14A	0.2463	0.3155	0.0918	0.062*	
H14B	0.3867	0.3523	0.1441	0.062*	
H14C	0.2657	0.3213	0.1818	0.062*	
C21	0.93204 (15)	−0.01030 (7)	0.25149 (8)	0.0249 (3)	
C22	0.83985 (17)	−0.03259 (8)	0.29494 (9)	0.0319 (3)	
H22	0.7394	−0.0424	0.2710	0.038*	
C23	0.89407 (18)	−0.04055 (9)	0.37329 (9)	0.0376 (4)	
H23	0.8297	−0.0556	0.4024	0.045*	
C24	1.04062 (18)	−0.02698 (8)	0.40987 (9)	0.0353 (4)	
H24	1.0763	−0.0326	0.4635	0.042*	
C25	1.13368 (18)	−0.00517 (8)	0.36723 (10)	0.0362 (4)	
H25	1.2345	0.0038	0.3913	0.043*	
C26	1.07971 (17)	0.00357 (8)	0.28947 (9)	0.0328 (3)	
H26	1.1442	0.0194	0.2609	0.039*	
C31	0.94616 (16)	−0.07369 (7)	0.11622 (9)	0.0270 (3)	
C32	0.9978 (2)	−0.12632 (8)	0.16371 (10)	0.0425 (4)	
H32	0.9980	−0.1242	0.2161	0.051*	
C33	1.0489 (2)	−0.18156 (9)	0.13569 (12)	0.0523 (5)	
H33	1.0840	−0.2169	0.1688	0.063*	
C34	1.0487 (2)	−0.18526 (9)	0.05995 (11)	0.0451 (4)	
H34	1.0836	−0.2232	0.0408	0.054*	
C35	0.99786 (18)	−0.13374 (8)	0.01163 (9)	0.0372 (4)	
H35	0.9971	−0.1365	−0.0408	0.045*	
C36	0.94815 (16)	−0.07838 (8)	0.03957 (9)	0.0308 (3)	
H36	0.9148	−0.0429	0.0062	0.037*	
C41	0.89248 (15)	0.27288 (7)	0.18795 (8)	0.0268 (3)	
C42	0.86834 (18)	0.29087 (8)	0.11127 (9)	0.0360 (4)	
H42	0.8192	0.2619	0.0723	0.043*	
C43	0.91518 (19)	0.35028 (9)	0.09168 (11)	0.0425 (4)	
H43	0.8969	0.3622	0.0395	0.051*	
C44	0.98901 (19)	0.39268 (8)	0.14815 (11)	0.0428 (4)	
H44	1.0223	0.4333	0.1346	0.051*	
C45	1.0138 (2)	0.37585 (8)	0.22366 (11)	0.0429 (4)	
H45	1.0639	0.4050	0.2622	0.051*	
C46	0.96594 (18)	0.31636 (8)	0.24391 (10)	0.0343 (3)	
H46	0.9834	0.3052	0.2963	0.041*	
C51	0.88289 (17)	0.19002 (7)	0.31191 (9)	0.0297 (3)	
C52	0.7990 (2)	0.22244 (9)	0.35306 (10)	0.0415 (4)	
H52	0.7145	0.2464	0.3270	0.050*	
C53	0.8384 (2)	0.21988 (11)	0.43235 (11)	0.0525 (5)	
H53	0.7803	0.2421	0.4601	0.063*	
C54	0.9611 (3)	0.18540 (10)	0.47112 (11)	0.0568 (6)	
H54	0.9873	0.1839	0.5253	0.068*	
C55	1.0442 (3)	0.15360 (10)	0.43126 (12)	0.0649 (6)	
H55	1.1289	0.1299	0.4577	0.078*	
C56	1.0059 (2)	0.15565 (9)	0.35211 (11)	0.0495 (5)	
H56	1.0647	0.1332	0.3250	0.059*	
O1	0.63781 (10)	0.07871 (4)	0.18809 (6)	0.0254 (2)	
P1	0.87456 (4)	0.001195 (17)	0.14751 (2)	0.02475 (9)	
P2	0.84253 (4)	0.189890 (18)	0.20739 (2)	0.02557 (9)	
S1	0.30570 (4)	0.064058 (19)	0.15779 (2)	0.03050 (10)	
C57	0.5549 (3)	0.08998 (11)	0.36408 (12)	0.0588 (5)	
H57A	0.5305	0.0567	0.3235	0.071*	
H57B	0.6410	0.1149	0.3574	0.071*	
Cl1	0.40384 (9)	0.14207 (3)	0.35502 (3)	0.07616 (19)	
Cl2	0.60096 (7)	0.05229 (3)	0.45321 (3)	0.06801 (17)	

### Atomic displacement parameters (Å^2^)

**Table:**

	*U*^11^	*U*^22^	*U*^33^	*U*^12^	*U*^13^	*U*^23^
C1	0.0241 (7)	0.0248 (7)	0.0251 (7)	−0.0002 (5)	0.0067 (5)	0.0043 (5)
C2	0.0296 (7)	0.0257 (7)	0.0280 (7)	−0.0009 (6)	0.0091 (6)	0.0003 (6)
C3	0.0308 (7)	0.0282 (7)	0.0247 (7)	−0.0043 (6)	0.0057 (6)	0.0014 (6)
C4	0.0249 (7)	0.0309 (7)	0.0253 (7)	−0.0040 (6)	0.0047 (6)	0.0032 (6)
C5	0.0248 (7)	0.0258 (7)	0.0227 (7)	0.0007 (5)	0.0059 (5)	0.0038 (5)
C6	0.0251 (7)	0.0229 (7)	0.0214 (6)	−0.0011 (5)	0.0045 (5)	0.0027 (5)
C7	0.0238 (7)	0.0282 (7)	0.0198 (6)	0.0004 (6)	0.0052 (5)	−0.0020 (5)
C8	0.0293 (7)	0.0249 (7)	0.0272 (7)	0.0013 (6)	0.0068 (6)	−0.0024 (6)
C9	0.0291 (7)	0.0298 (7)	0.0293 (8)	0.0061 (6)	0.0070 (6)	−0.0013 (6)
C10	0.0233 (7)	0.0352 (8)	0.0322 (8)	0.0030 (6)	0.0075 (6)	−0.0019 (6)
C11	0.0244 (7)	0.0295 (7)	0.0246 (7)	−0.0011 (6)	0.0074 (6)	−0.0007 (6)
C12	0.0246 (7)	0.0260 (7)	0.0191 (6)	0.0031 (5)	0.0051 (5)	0.0008 (5)
C13	0.0369 (9)	0.0360 (9)	0.0406 (9)	−0.0076 (7)	0.0107 (7)	−0.0098 (7)
C14	0.0365 (9)	0.0333 (9)	0.0520 (11)	0.0091 (7)	0.0092 (8)	−0.0033 (7)
C21	0.0251 (7)	0.0226 (7)	0.0275 (7)	−0.0001 (5)	0.0077 (6)	0.0006 (5)
C22	0.0248 (7)	0.0389 (8)	0.0326 (8)	−0.0013 (6)	0.0083 (6)	0.0037 (7)
C23	0.0347 (8)	0.0482 (10)	0.0329 (8)	0.0002 (7)	0.0143 (7)	0.0074 (7)
C24	0.0381 (8)	0.0388 (9)	0.0275 (8)	0.0037 (7)	0.0056 (7)	0.0012 (7)
C25	0.0290 (8)	0.0400 (9)	0.0366 (9)	−0.0044 (7)	0.0028 (7)	−0.0006 (7)
C26	0.0278 (7)	0.0366 (8)	0.0344 (8)	−0.0057 (6)	0.0090 (6)	0.0021 (7)
C31	0.0239 (7)	0.0295 (7)	0.0295 (7)	−0.0012 (6)	0.0100 (6)	0.0010 (6)
C32	0.0623 (12)	0.0354 (9)	0.0346 (9)	0.0130 (8)	0.0213 (8)	0.0060 (7)
C33	0.0747 (14)	0.0362 (10)	0.0512 (11)	0.0197 (9)	0.0256 (10)	0.0075 (8)
C34	0.0497 (11)	0.0378 (9)	0.0529 (11)	0.0042 (8)	0.0222 (9)	−0.0104 (8)
C35	0.0331 (8)	0.0480 (10)	0.0332 (8)	−0.0062 (7)	0.0133 (7)	−0.0104 (7)
C36	0.0262 (7)	0.0366 (8)	0.0295 (8)	−0.0028 (6)	0.0070 (6)	0.0014 (6)
C41	0.0230 (7)	0.0286 (7)	0.0307 (8)	0.0013 (6)	0.0103 (6)	−0.0018 (6)
C42	0.0326 (8)	0.0440 (9)	0.0329 (8)	−0.0032 (7)	0.0109 (7)	0.0005 (7)
C43	0.0360 (9)	0.0503 (10)	0.0437 (10)	0.0030 (8)	0.0146 (8)	0.0166 (8)
C44	0.0377 (9)	0.0320 (8)	0.0648 (12)	0.0037 (7)	0.0244 (9)	0.0092 (8)
C45	0.0466 (10)	0.0309 (8)	0.0563 (11)	−0.0075 (7)	0.0224 (9)	−0.0110 (8)
C46	0.0391 (9)	0.0325 (8)	0.0340 (8)	−0.0034 (7)	0.0141 (7)	−0.0045 (6)
C51	0.0310 (7)	0.0262 (7)	0.0288 (7)	−0.0041 (6)	0.0022 (6)	0.0022 (6)
C52	0.0394 (9)	0.0540 (11)	0.0308 (8)	−0.0005 (8)	0.0084 (7)	0.0023 (8)
C53	0.0611 (12)	0.0645 (13)	0.0334 (10)	−0.0123 (10)	0.0150 (9)	−0.0037 (9)
C54	0.0847 (16)	0.0486 (11)	0.0274 (9)	−0.0151 (11)	−0.0030 (9)	0.0046 (8)
C55	0.0803 (16)	0.0536 (12)	0.0421 (11)	0.0176 (11)	−0.0182 (11)	0.0022 (10)
C56	0.0538 (11)	0.0435 (10)	0.0405 (10)	0.0141 (9)	−0.0069 (8)	−0.0034 (8)
O1	0.0243 (5)	0.0226 (5)	0.0271 (5)	0.0010 (4)	0.0027 (4)	−0.0005 (4)
P1	0.02381 (18)	0.02421 (18)	0.02701 (19)	−0.00075 (14)	0.00797 (14)	0.00316 (14)
P2	0.02309 (18)	0.02554 (19)	0.02756 (19)	0.00042 (14)	0.00558 (14)	−0.00296 (14)
S1	0.02473 (18)	0.0316 (2)	0.0379 (2)	−0.00240 (14)	0.01310 (15)	−0.00110 (16)
C57	0.0696 (14)	0.0653 (13)	0.0483 (12)	−0.0136 (11)	0.0277 (11)	0.0025 (10)
Cl1	0.1172 (6)	0.0525 (3)	0.0529 (3)	0.0151 (3)	0.0113 (3)	0.0040 (2)
Cl2	0.0751 (4)	0.0842 (4)	0.0416 (3)	0.0195 (3)	0.0094 (3)	0.0037 (3)

### Geometric parameters (Å, º)

**Table:**

C1—C6	1.393 (2)	C26—H26	0.9500
C1—C2	1.405 (2)	C31—C32	1.395 (2)
C1—P1	1.8492 (14)	C31—C36	1.399 (2)
C2—C3	1.395 (2)	C31—P1	1.8345 (15)
C2—H2	0.9500	C32—C33	1.384 (2)
C3—C4	1.384 (2)	C32—H32	0.9500
C3—C13	1.513 (2)	C33—C34	1.375 (3)
C4—C5	1.392 (2)	C33—H33	0.9500
C4—H4	0.9500	C34—C35	1.384 (3)
C5—C6	1.3953 (19)	C34—H34	0.9500
C5—S1	1.7601 (14)	C35—C36	1.380 (2)
C6—O1	1.3996 (16)	C35—H35	0.9500
C7—C12	1.3961 (19)	C36—H36	0.9500
C7—C8	1.4035 (19)	C41—C46	1.397 (2)
C7—P2	1.8489 (14)	C41—C42	1.400 (2)
C8—C9	1.399 (2)	C41—P2	1.8367 (15)
C8—H8	0.9500	C42—C43	1.381 (2)
C9—C10	1.387 (2)	C42—H42	0.9500
C9—C14	1.507 (2)	C43—C44	1.389 (3)
C10—C11	1.391 (2)	C43—H43	0.9500
C10—H10	0.9500	C44—C45	1.373 (3)
C11—C12	1.3983 (19)	C44—H44	0.9500
C11—S1	1.7610 (15)	C45—C46	1.391 (2)
C12—O1	1.3966 (16)	C45—H45	0.9500
C13—H13A	0.9800	C46—H46	0.9500
C13—H13B	0.9800	C51—C52	1.387 (2)
C13—H13C	0.9800	C51—C56	1.389 (2)
C14—H14A	0.9800	C51—P2	1.8349 (16)
C14—H14B	0.9800	C52—C53	1.390 (2)
C14—H14C	0.9800	C52—H52	0.9500
C21—C22	1.389 (2)	C53—C54	1.380 (3)
C21—C26	1.406 (2)	C53—H53	0.9500
C21—P1	1.8381 (15)	C54—C55	1.360 (3)
C22—C23	1.390 (2)	C54—H54	0.9500
C22—H22	0.9500	C55—C56	1.387 (3)
C23—C24	1.390 (2)	C55—H55	0.9500
C23—H23	0.9500	C56—H56	0.9500
C24—C25	1.381 (2)	C57—Cl2	1.745 (2)
C24—H24	0.9500	C57—Cl1	1.751 (2)
C25—C26	1.382 (2)	C57—H57A	0.9900
C25—H25	0.9500	C57—H57B	0.9900
			
C6—C1—C2	117.04 (13)	C32—C31—P1	124.33 (12)
C6—C1—P1	119.63 (11)	C36—C31—P1	117.76 (11)
C2—C1—P1	123.24 (11)	C33—C32—C31	120.99 (16)
C3—C2—C1	122.92 (13)	C33—C32—H32	119.5
C3—C2—H2	118.5	C31—C32—H32	119.5
C1—C2—H2	118.5	C34—C33—C32	120.07 (17)
C4—C3—C2	118.47 (13)	C34—C33—H33	120.0
C4—C3—C13	120.31 (14)	C32—C33—H33	120.0
C2—C3—C13	121.22 (14)	C33—C34—C35	120.08 (16)
C3—C4—C5	120.04 (13)	C33—C34—H34	120.0
C3—C4—H4	120.0	C35—C34—H34	120.0
C5—C4—H4	120.0	C36—C35—C34	119.97 (15)
C4—C5—C6	120.73 (13)	C36—C35—H35	120.0
C4—C5—S1	119.29 (11)	C34—C35—H35	120.0
C6—C5—S1	119.92 (11)	C35—C36—C31	120.98 (15)
C1—C6—C5	120.74 (13)	C35—C36—H36	119.5
C1—C6—O1	117.41 (12)	C31—C36—H36	119.5
C5—C6—O1	121.77 (12)	C46—C41—C42	118.26 (14)
C12—C7—C8	117.39 (13)	C46—C41—P2	124.34 (12)
C12—C7—P2	118.59 (10)	C42—C41—P2	117.07 (12)
C8—C7—P2	124.00 (11)	C43—C42—C41	120.73 (16)
C9—C8—C7	122.37 (14)	C43—C42—H42	119.6
C9—C8—H8	118.8	C41—C42—H42	119.6
C7—C8—H8	118.8	C42—C43—C44	120.15 (16)
C10—C9—C8	118.53 (13)	C42—C43—H43	119.9
C10—C9—C14	119.65 (14)	C44—C43—H43	119.9
C8—C9—C14	121.81 (14)	C45—C44—C43	119.93 (16)
C9—C10—C11	120.66 (13)	C45—C44—H44	120.0
C9—C10—H10	119.7	C43—C44—H44	120.0
C11—C10—H10	119.7	C44—C45—C46	120.29 (16)
C10—C11—C12	119.89 (13)	C44—C45—H45	119.9
C10—C11—S1	119.63 (11)	C46—C45—H45	119.9
C12—C11—S1	120.42 (11)	C45—C46—C41	120.62 (16)
C7—C12—O1	117.70 (12)	C45—C46—H46	119.7
C7—C12—C11	121.12 (13)	C41—C46—H46	119.7
O1—C12—C11	121.11 (12)	C52—C51—C56	118.19 (16)
C3—C13—H13A	109.5	C52—C51—P2	124.20 (12)
C3—C13—H13B	109.5	C56—C51—P2	117.61 (13)
H13A—C13—H13B	109.5	C51—C52—C53	120.12 (18)
C3—C13—H13C	109.5	C51—C52—H52	119.9
H13A—C13—H13C	109.5	C53—C52—H52	119.9
H13B—C13—H13C	109.5	C54—C53—C52	120.7 (2)
C9—C14—H14A	109.5	C54—C53—H53	119.7
C9—C14—H14B	109.5	C52—C53—H53	119.7
H14A—C14—H14B	109.5	C55—C54—C53	119.59 (18)
C9—C14—H14C	109.5	C55—C54—H54	120.2
H14A—C14—H14C	109.5	C53—C54—H54	120.2
H14B—C14—H14C	109.5	C54—C55—C56	120.28 (19)
C22—C21—C26	117.78 (14)	C54—C55—H55	119.9
C22—C21—P1	124.39 (11)	C56—C55—H55	119.9
C26—C21—P1	117.83 (11)	C55—C56—C51	121.12 (19)
C21—C22—C23	120.19 (14)	C55—C56—H56	119.4
C21—C22—H22	119.9	C51—C56—H56	119.4
C23—C22—H22	119.9	C12—O1—C6	117.53 (10)
C24—C23—C22	121.29 (15)	C31—P1—C21	100.06 (7)
C24—C23—H23	119.4	C31—P1—C1	100.04 (6)
C22—C23—H23	119.4	C21—P1—C1	102.95 (6)
C25—C24—C23	119.08 (15)	C51—P2—C41	101.66 (7)
C25—C24—H24	120.5	C51—P2—C7	100.36 (7)
C23—C24—H24	120.5	C41—P2—C7	101.89 (6)
C24—C25—C26	119.79 (15)	C5—S1—C11	98.28 (7)
C24—C25—H25	120.1	Cl2—C57—Cl1	111.22 (11)
C26—C25—H25	120.1	Cl2—C57—H57A	109.4
C25—C26—C21	121.85 (14)	Cl1—C57—H57A	109.4
C25—C26—H26	119.1	Cl2—C57—H57B	109.4
C21—C26—H26	119.1	Cl1—C57—H57B	109.4
C32—C31—C36	117.91 (14)	H57A—C57—H57B	108.0
			
C6—C1—C2—C3	1.0 (2)	C46—C41—C42—C43	−0.4 (2)
P1—C1—C2—C3	−175.58 (11)	P2—C41—C42—C43	−174.05 (13)
C1—C2—C3—C4	−1.8 (2)	C41—C42—C43—C44	0.9 (2)
C1—C2—C3—C13	178.82 (14)	C42—C43—C44—C45	−0.8 (3)
C2—C3—C4—C5	0.4 (2)	C43—C44—C45—C46	0.3 (3)
C13—C3—C4—C5	179.78 (14)	C44—C45—C46—C41	0.2 (3)
C3—C4—C5—C6	1.7 (2)	C42—C41—C46—C45	−0.1 (2)
C3—C4—C5—S1	−175.53 (11)	P2—C41—C46—C45	173.01 (13)
C2—C1—C6—C5	1.2 (2)	C56—C51—C52—C53	−0.2 (3)
P1—C1—C6—C5	177.91 (10)	P2—C51—C52—C53	−179.86 (14)
C2—C1—C6—O1	178.01 (12)	C51—C52—C53—C54	0.2 (3)
P1—C1—C6—O1	−5.27 (17)	C52—C53—C54—C55	0.0 (3)
C4—C5—C6—C1	−2.6 (2)	C53—C54—C55—C56	−0.1 (3)
S1—C5—C6—C1	174.68 (11)	C54—C55—C56—C51	0.1 (3)
C4—C5—C6—O1	−179.26 (12)	C52—C51—C56—C55	0.0 (3)
S1—C5—C6—O1	−2.00 (18)	P2—C51—C56—C55	179.73 (17)
C12—C7—C8—C9	−0.8 (2)	C7—C12—O1—C6	−145.15 (12)
P2—C7—C8—C9	−178.94 (11)	C11—C12—O1—C6	37.94 (17)
C7—C8—C9—C10	1.0 (2)	C1—C6—O1—C12	145.19 (12)
C7—C8—C9—C14	−179.24 (14)	C5—C6—O1—C12	−38.02 (17)
C8—C9—C10—C11	0.4 (2)	C32—C31—P1—C21	11.81 (16)
C14—C9—C10—C11	−179.35 (14)	C36—C31—P1—C21	−168.59 (11)
C9—C10—C11—C12	−2.0 (2)	C32—C31—P1—C1	−93.41 (15)
C9—C10—C11—S1	175.18 (12)	C36—C31—P1—C1	86.19 (12)
C8—C7—C12—O1	−177.71 (12)	C22—C21—P1—C31	−98.97 (13)
P2—C7—C12—O1	0.51 (17)	C26—C21—P1—C31	80.13 (12)
C8—C7—C12—C11	−0.8 (2)	C22—C21—P1—C1	3.88 (14)
P2—C7—C12—C11	177.42 (10)	C26—C21—P1—C1	−177.02 (12)
C10—C11—C12—C7	2.2 (2)	C6—C1—P1—C31	174.71 (11)
S1—C11—C12—C7	−174.93 (10)	C2—C1—P1—C31	−8.79 (13)
C10—C11—C12—O1	178.99 (13)	C6—C1—P1—C21	71.84 (12)
S1—C11—C12—O1	1.87 (18)	C2—C1—P1—C21	−111.66 (12)
C26—C21—C22—C23	0.0 (2)	C52—C51—P2—C41	71.69 (15)
P1—C21—C22—C23	179.12 (12)	C56—C51—P2—C41	−107.97 (14)
C21—C22—C23—C24	−0.4 (3)	C52—C51—P2—C7	−32.88 (15)
C22—C23—C24—C25	0.0 (3)	C56—C51—P2—C7	147.46 (13)
C23—C24—C25—C26	0.8 (2)	C46—C41—P2—C51	9.70 (14)
C24—C25—C26—C21	−1.2 (2)	C42—C41—P2—C51	−177.11 (12)
C22—C21—C26—C25	0.8 (2)	C46—C41—P2—C7	113.06 (13)
P1—C21—C26—C25	−178.36 (12)	C42—C41—P2—C7	−73.75 (12)
C36—C31—C32—C33	−0.3 (3)	C12—C7—P2—C51	−88.24 (12)
P1—C31—C32—C33	179.32 (15)	C8—C7—P2—C51	89.85 (13)
C31—C32—C33—C34	−0.2 (3)	C12—C7—P2—C41	167.38 (11)
C32—C33—C34—C35	0.1 (3)	C8—C7—P2—C41	−14.54 (13)
C33—C34—C35—C36	0.5 (3)	C4—C5—S1—C11	−150.31 (12)
C34—C35—C36—C31	−1.0 (2)	C6—C5—S1—C11	32.39 (12)
C32—C31—C36—C35	0.9 (2)	C10—C11—S1—C5	150.43 (12)
P1—C31—C36—C35	−178.76 (12)	C12—C11—S1—C5	−32.45 (13)

## References

[bb1] Bruker (2005). *APEX2* and *SAINT-Plus* Bruker AXS Inc., Madison, Wisconsin, USA.

[bb2] Goertz, W., Keim, W., Vogt, D., Englert, U., Boele, M. D. K., van der Veen, L. A., Kamer, P. C. J. & van Leeuwen, P. W. N. M. (1998). *J. Chem. Soc. Dalton Trans.* pp. 2981–2988.

[bb3] Hillebrand, S., Bruckmann, J., Kruger, C. & Haenel, M. W. (1995). *Tetrahedron Lett.* **36**, 75–78.

[bb4] Kranenburg, M., Vanderburgt, Y. E. M., Kamer, P. C. J., van Leeuwen, P. W. N. M., Goubitz, K. & Fraanje, J. (1995). *Organometallics*, **14**, 3081–3089.

[bb5] Marimuthu, T., Bala, M. D. & Friedrich, H. B. (2008). *Acta Cryst.* E**64**, o711. 10.1107/S1600536808006648 PMC2960967 21202102

[bb6] Sheldrick, G. M. (2008). *Acta Cryst.* A**64**, 112–122. 10.1107/S0108767307043930 18156677

